# Supplementary material for: Histotripsy-Initiated Immune Response Synergizes with Chemotherapy in a Neuroblastoma Murine Model
Source: Cancers (Basel). 2026 Apr 15;18(8):1249. doi: 10.3390/cancers18081249 (PMC13114721; doi:10.3390/cancers18081249)
Supplement: Supplementary file 1 [file cancers-18-01249-s001.zip › Supplementary FiguresTables_V2.pdf]

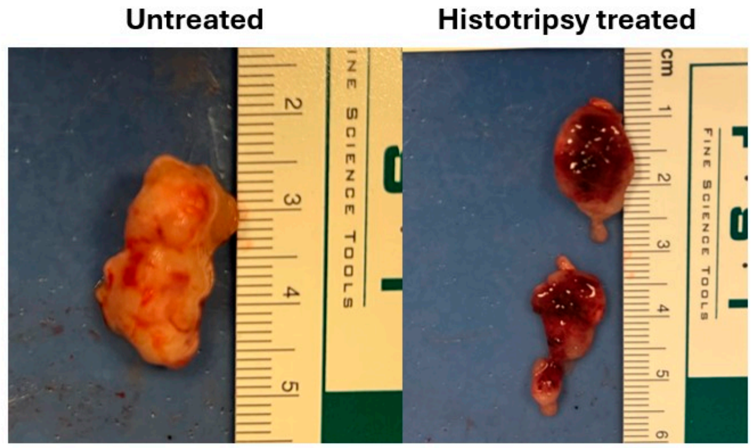

Supplementary Figure 1. Representative images of untreated tumors and tumors treated with histotripsy (80% of tumor targeted). Treated tumor has been sectioned in half.

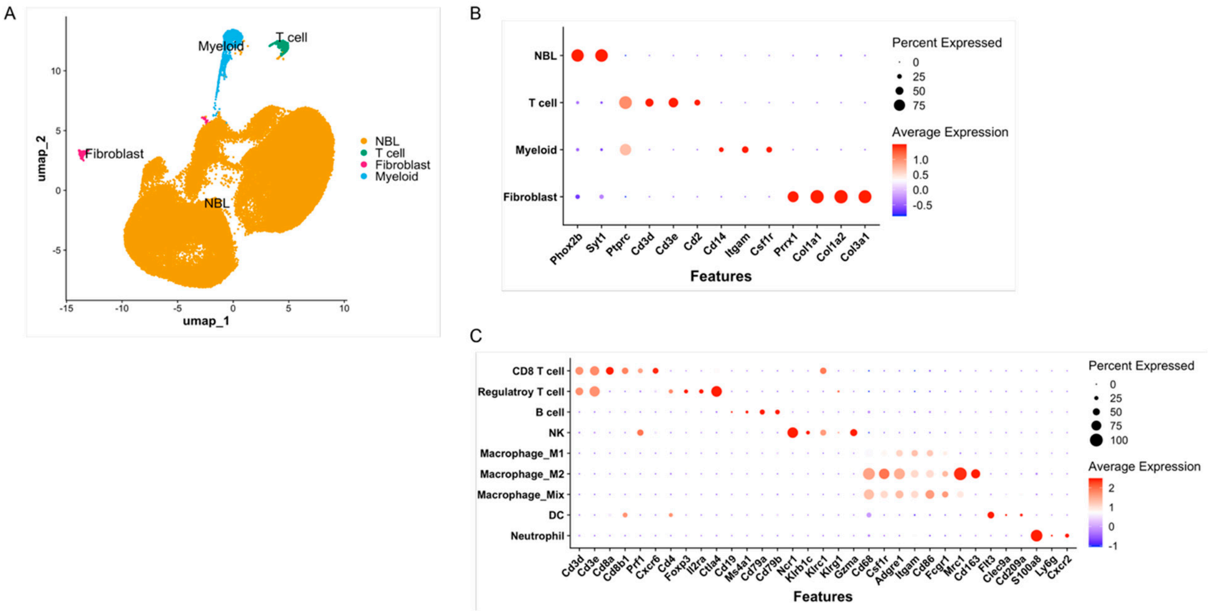

Supplementary Figure 2. scRNA-seq UMAP and marker gene expression across treatment conditions. **A,** Annotated integrated uniform manifold approximation and projection (UMAP) of scRNA-seq from untreated (n = 3), histotripsy-treated (n = 2), and contralateral tumors (n = 2). Four major cell types were identified: neuroblastoma tumor cells (NBL), T cells, myeloid cells, and fibroblasts. **B,** Dotplot of key marker gene expression used for annotation in A. **C,** Dotplot of key marker gene expression used for annotation in Fig. 3A.

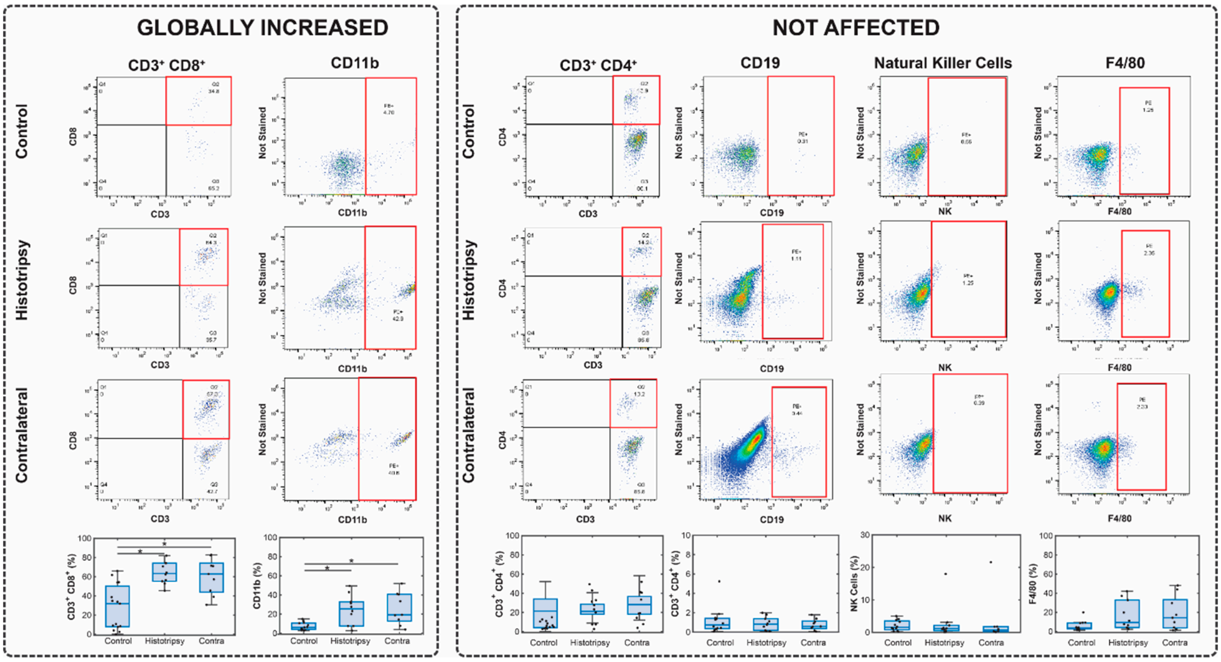

Supplementary Figure 3. Flow cytometry analysis indicated histotripsy elicited both innate and adaptive immune responses. The percentage of $CD3^+CD8^+$ T cells was increased in histotripsy-treated (n=10) and contralateral tumors (n = 9) compared with untreated controls (n = 15) ($p < 0.05$ for both comparisons, Welch's ANOVA with Games-Howell post hoc). Similarly, $CD11b^+$ cells were increased in histotripsy-treated (n=10) and contralateral tumors (n=9) relative to untreated controls (n=13) ($p < 0.05$ for both comparisons, Welch's ANOVA with Games-Howell post hoc). No significant differences were observed between histotripsy-treated and contralateral tumors for either $CD3^+CD8^+$ T cells or $CD11b^+$ cells. No significant differences were observed across experimental groups for the other markers assessed with flow cytometry.

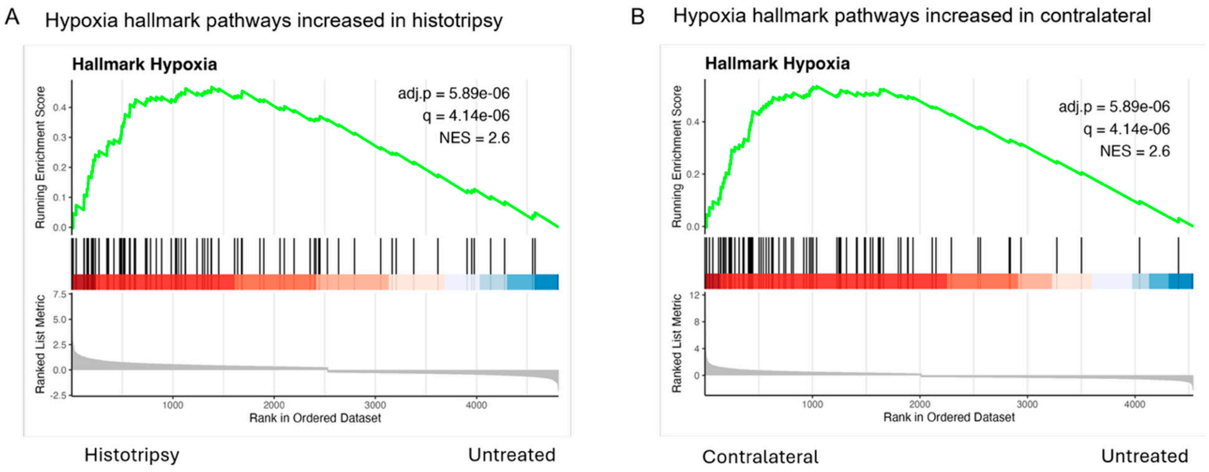

Supplementary Figure 4. Hypoxia pathway is enriched in histotripsy-treated and contralateral tumors. **A,** GSEA enrichment plot for hypoxia hallmark pathway for histotripsy-treated tumors compared with control. **B,** GSEA enrichment plot for hypoxia hallmark pathway in contralateral tumors compared with control.

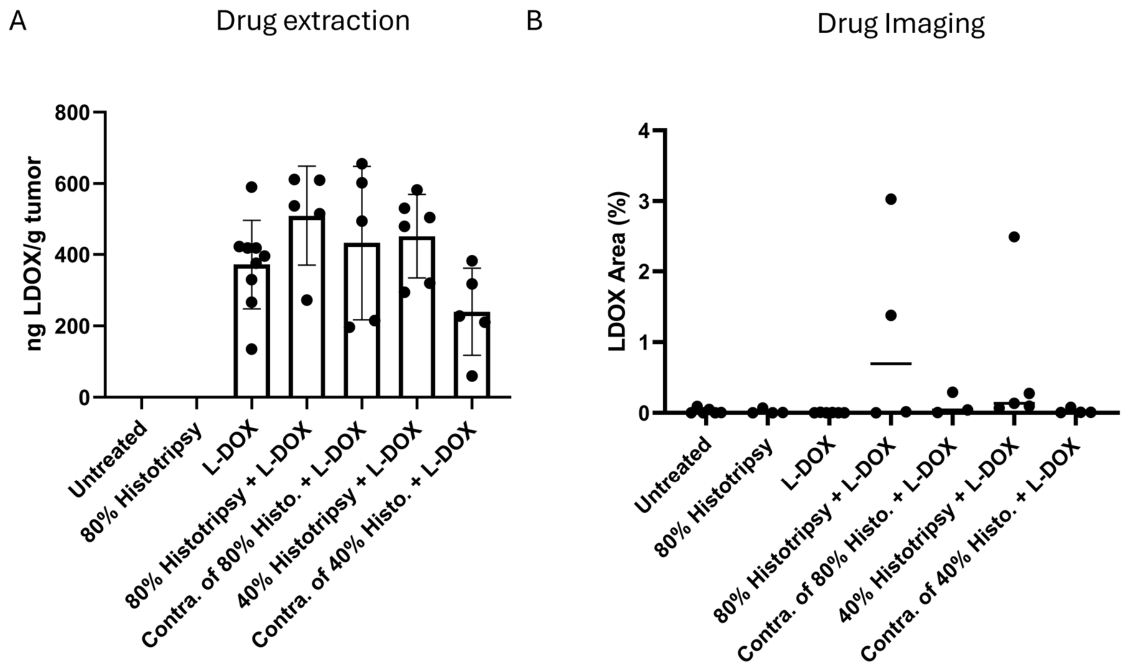

Supplementary Figure 5. Histotripsy does not increase short-term uptake or distribution of L-DOX in tumors for 40% to 80% tumor coverage. **A**, Quantification of L-DOX extracted from tumors 24 hours after treatment (untreated, n = 8; 80% histotripsy, n = 3; L-DOX, n = 9; 80% histotripsy + L-DOX, n = 5; contralateral of 80% histotripsy + L-DOX, n = 5; 40% histotripsy + L-DOX, n = 6; contralateral of 40% histotripsy + L-DOX, n = 5). Tumors receiving combined histotripsy + L-DOX, regardless of total tumor coverage by histotripsy, showed no significant increase in L-DOX levels compared to L-DOX alone (unpaired t-test, $p > 0.05$). Contralateral tumors similarly showed no significant difference relative to L-DOX alone (unpaired t-test, $p > 0.05$). **B**, Assessment of the area of L-DOX distribution within tumors (untreated, n = 6; histotripsy, n = 4; L-DOX, n = 4; 80% histotripsy + L-DOX, n = 3; contralateral of 80% histotripsy + L-DOX, n = 5; 40% histotripsy + L-DOX, n = 5; contralateral of 40% histotripsy + L-DOX, n = 4). While individual histotripsy-treated tumors showed strong L-DOX coverage, there were no overall differences between groups (one-way ANOVA, $p > 0.05$).

Use	Antibody	Fluorescent dye	Clone	Dilution *	Catalog number	Company
Flow	CD3	PE	17A2	1:50	100206	Biolegend
Flow	CD45.2	FITC	104	1:50	109806	Biolegend
Flow	CD45	BUV395	30-F11	1:50	564279	BD Bioscience
Flow	CD4	APC	RM4-5	1:50	100516	Biolegend
Flow	CD8a	APC	53-6.7	1:50	100712	Biolegend
Flow	CD19	PE	6D5	1:50	115508	Biolegend
Flow	CD8	Pacific Blue	53-6.7	1:50	100725	Biolegend
Flow	CD11b	PE	M1/70	1:50	55311	Biolegend
Flow	CD11c	PE	N418	1:50	117308	Biolegend
Flow	F4/80	eFluor570	BM8	1:50	41-4801-82	Invitrogen
Flow	Granzyme B	FITC	QA1602	1:50	372206	Biolegend
Flow	IFN-gamma	FITC	505806	1:50	B391662	Biolegend
Flow<br>Flow	I-A/I-E<br>(MHC class II)	APC	M5/114.15.2	1:50	107614	Biolegend
Flow	Ki67	Pacific Blue	164.8	1:50	65422	Biolegend
Flow	Perforin	FITC	516009A	1:50	154310	Biolegend
Flow<br>Flow	Fc Block<br>(CD16/32)	none	93	1:50	101320	Biolegend

*mL Ab/50 mL diluent with 3-6x10^6 cells

Supplementary Table 1. Panel of antibodies used to stain single-cell tumor suspensions for flow cytometry.

Supplemental Video 1. Standard ultrasound image and passive image of bubble cloud generation during the application of histotripsy to the flank Neuro-2a tumor.
